# Genome Sequence of a *Lactococcus lactis* Strain Isolated from Salmonid Intestinal Microbiota

**DOI:** 10.1128/genomeA.00881-16

**Published:** 2016-08-25

**Authors:** Rafael Opazo, Felipe Gajardo, Mauricio Ruiz, Jaime Romero

**Affiliations:** a Laboratorio de Biotecnología, Instituto de Nutrición y Tecnología de los Alimentos, Universidad de Chile, Macul, Santiago, Chile; b Departamento de Desarrollo, Helios Innovation, Matucana, Casablanca, Chile

## Abstract

*Lactococcus lactis* is a common inhabitant of the intestinal microbiota of salmonids, especially those in aquaculture systems. Here, we present a genome sequence of a *Lactococcus lactis* strain isolated from the intestinal contents of rainbow trout reared in Chile.

## GENOME ANNOUNCEMENT

Currently, it is generally recognized that the gastrointestinal microbiota of animals serves several functions, including nutrition, development, immunity, and xenobiotic metabolism (1). Using molecular approaches to examine the gut microbiota of farmed Atlantic salmon and rainbow trout, several authors reported that lactic acid bacteria (LAB) were among the predominant bacterial groups; hence, *Lactococcus* and *Lactobacillus* were observed in most of the molecular profiles derived from samples from those fish (1).

Strains of *Lactococcus* have been proposed to be probiotic; some studies have suggested that protection and stimulation of the immune system are conferred by several strains of this genus. Furthermore, the immunomodulatory properties of *Lactococcus lactis* strains have been shown recently, for example, in the context of soy-induced enteritis in salmon (2). These authors showed that the addition of *Lactococcus lactis* strain RBT018 to the soybean meal diet decreased the inflammation parameters in the histological examination compared to soybean meal diet without the probiotic.

The bacterial genome was sequenced using the Ion Torrent PGM platform with mate-paired-end 3-kbp span library for each isolate. The data were quality trimmed using Prinseq with a Phred score of 15, and sequencing errors were corrected using the software Pollux and subsequently assembled with Celera Assembler version 8.3. The assembled sequence was annotated by the National Center for Biotechnology Information (NCBI) Prokaryotic Genome Annotation Pipeline (PGAP). The resulting genome assembly of RBT018 has 79 contigs, comprising 2,479,393 bp. The estimated G+C content of the assembled RBT018 genome is 35.4%. The annotation of the RBT018 assembly contains 2,630 genes, including 2,419 coding DNA sequences (CDSs), 123 pseudogenes, 23 rRNA genes (5S, 16S, and 23S), 64 tRNA genes, one ribozyme gene (RNase P), and 110 frameshifted genes. The genome size, G+C content, number of predicted genes, and RNA coding genes are comparable to those of the other published *Lactococcus* strains (3). RBT018 was compared with the probiotic strain *Lactococcus lactis* CV56 (accession numbers CP002365 to CP002370) using InParanoid8 (4). Some of the common genes in both strains that could be related to probiotic activity were collagen adhesin (accession no. OAZ16633.1), two bacteriocins (accession numbers OAZ16685.1 and OAZ16145.1), and bacteriocin ABC transporter ATP-binding protein (accession no. OAZ16685.1). After a BLASTn search (with default parameters) against the Virulence Factors Database (VFDB [http://www.mgc.ac.cn/VFs/]), none of the genes in the RBT018 genome were found to be similar to virulence genes in VFDB.

### Accession number(s).

The genome sequence was deposited in DDBJ/EMBL/GenBank under the accession no. JCOB00000000. The version described in this paper is the first version.
